# Intelligent routing for human activity recognition in wireless body area networks

**DOI:** 10.1038/s41598-025-12114-3

**Published:** 2025-07-29

**Authors:** Enas Selem Elmosallamy, Mohammed F. Soliman

**Affiliations:** 1 Information Technology Department, Faculty of Computer and Information Suez University, Suez, 43221 Egypt; 2 Electrical and Computer Engineering Department, Iowa State University, Ames, 50011 United States

**Keywords:** Machine learning, Network topology

## Abstract

Human activity recognition (HAR), driven by machine learning techniques, offer the detection of diverse activities such as walking, running, and more. Considering the dynamic nature, limited energy and mobility of wireless body area networks (WBANs), HAR can play a significant role in enhancing WBANs performance. This paper genuinely bridges HAR’s activity recognition capability using machine learning to develop a novel WBAN routing decisions adoptively. Being optimum in power consumption, we employed Random Forest classification algorithm for activity recognition. The resulted system holds great promise for optimizing routing decisions, improving energy efficiency, and enhancing the overall performance of WBANs in healthcare and related domains. To evaluate the performance of the proposed protocol, we have measured various performance metrics, including energy consumption, throughput, and the number of dead nodes. The results have been compared with mobTHE protocol to demonstrate the effectiveness of our HAR based Routing protocol.

## Introduction

Human Activity Recognition (HAR) has attracted a great interest in recent years due to its use in various applications ranging from assistive living^1,2^, nursing home^3^, health monitoring^4^, rehabilitation activities^5,6^, surveillance^7^, and human-computer interaction^8^...etc. The main goal of HAR is recognizing and classifying the human physical activities based on data collected from sensor nodes. These activities such as running, jumping, walking, and sitting, can be recognized through changes in the entire body^9^. Some activities are identified by the movement of specific body parts, such as making hand gestures. Other activities involve interaction with objects, like cooking in the kitchen. HAR also includes the detection of abnormal activities, such as sudden falls^10,11^. Basically, HAR can be classified into two types based on the collected data: vision-based HAR^12,13^ and sensor-based HAR^14^. Vision-based techniques analyze camera data in video or image formats, while sensor-based systems interpret data from sensor nodes such as accelerometers, gyroscopes, radar, and magnetometers in the form of time series. In this paper, we utilize the sensor-based HAR as it is the most suitable type for recognizing human activates in WBAN.

WBAN consists of teeny, battery-powered, and lightweight sensor nodes that monitor physiological data^15^. These sensor nodes can be either implanted (in-body) or worn (on-body). They wirelessly communicate with a central monitoring system, which can be either on the person’s body (sink node) or at a remote location, such as a hospital. Each sensor node in a WBAN senses vital signs of human body and transmits it wirelessly to the sink node. The sink node then forwards the collected data to a gateway for further processing and analysis. Any abnormalities detected in the data are immediately sent to a medical center for necessary treatment. Utilizing WBANs for health monitoring enable continuous and real-time monitoring of human’ vital signs and activities which result in the early detection of health issues and enhances patient outcomes. the transmission of these vital signs from sensor nodes to sink node may be done in one-hop when the nodes are in close proximity. However, to reduce energy consumption multi-hops may be used where the source node transmit its data to the sink node via one or more relay sensor nodes. This introduces the need for routing protocols specifically designed for WBANs.

Routing in WBANs is a challenging due to the dynamic nature of the human body which exert various activities that causes high mobility of body parts leads to frequent changes in the network topology, making it difficult to maintain stable wireless links and may cause topological disconnection. Additionally, the limited power and processing capabilities of battery powered sensor nodes require energy-efficient routing protocols to extend the sensor nodes’ lifetime and ensure reliable data communications.

To tackle these issues, Adaptive routing protocols have been proposed which characterized by the ability of the network to dynamically adjust the routing paths based on changing network conditions, such as node mobility, link quality, node energy levels, and traffic patterns. In this paper, we utilize human activity recognition in adaptive routing in WBANs to adjust routing decisions based on the recognized activity. The routing decisions differentiate between resting activities (e.g., sitting or lying down), normal activities (e.g., walking), exercising Activity(e.g., jogging or cycling) and critical health events (e.g., a fall) to ensure timely and accurately responses. Also, the proposed protocol dynamically adjust routing path based on the current activity which overcome the frequent change of network topology which resulted in disconnection among sensor nodes. Moreover, the designed protocol take into account the limited energy resources of WBAN, so that we choose the least power consumption machine learning algorithm to be used in the training process of human activity recognition. The rest of the paper is organized as follows. In section 3, related research is reviewed, while in section 4, Physical activity Recognition Model is introduced. In section 5, The description of PAMAP2 dataset , also in section 6, Model Training and Evaluation is discussed. Impact of Human Activity Recognition on Routing Performance is presented in section 7, System model is described in section 8, proposed model is introduced in section 9, Simulation Result is explained in section 10, and the conclusions are outlined in section 11.

## Related research

The integration between Human Activity Recognition (HAR) and Wireless Body Area Networks (WBANs) is a growing area of research that aims to identify and classify human activities using data from sensor nodes worn on the body. There are various types of sensor nodes to collect data for HAR in WBANs. Accelerometers and gyroscopes are commonly used because they can effectively capture movements and orientations. These sensor nodes provide data on activities like walking, running, and sitting. WBANs allow multiple sensor nodes to work together to provide more accurate activity recognition. Studies have shown that using multiple sensor nodes, such as those worn on different parts of the body, can capture a more complete picture of a person’s movements. This leads to better recognition accuracy compared to using a single sensor, this what we achieve in this work. Another type of activity recognition is Vision-based HAR, in this type cameras are used to monitor activities. This type of HAR recognize actions by analyzing video or image data. This analysis of videos or images need more processing power and consume more energy, therefore,this type of HAR is not appropriate for using in WBANs due to its limited energy resources . HAR^16–18^ depends on machine learning algorithms, ranging from basic techniques to more advanced ones. Now we will review some of the machine learning algorithms that are used in HAR. Support Vector Machines (SVMs)^19,20^, Hidden Markov Models (HMMs)^21,22^, Decision Trees^23–26^, Recurrent Neural Networks (RNNs)^27,28^, Reinforcement Learning (RL)^29,30^. SVMs can work well in cases where there is a clear difference between activities and the data has many features. However, SVMs can be slow, require a lot of processing power, which can be difficult for small, battery-powered devices like those in WBANs. On the other hand, HHMs are working well at handling sequences and time-based data. However, they can’t capture the complex changes in WBAN sensor data well, and they rely on set rules for transitions, which can be limiting. Decision Trees make decisions by asking a series of simple questions, which makes their operation easy to follow. However they suffer from overfiting which make them unstable, with small changes in data leading to very different results.

While Support Vector Machines, Hidden Markov Models, Decision Trees, Recurrent Neural Networks, and Reinforcement Learning have all been important for recognizing activities. Random Forests are considered the optimal algorithm for energy constrained WBAN because it has the lowest computational complexity and is characterized by its simplicity, reliability, and can handle various situations well. By comparing the computational complexity of random forests and aforementioned algorithms, it can be seen from Table 1 that Descion tree, and Random Forests have the lowest computational complexity but decision tree can’t perform well under heavy traffic as it suffers from overfitting which means that Random Forests are considered the best choice for activity recognition in WBANs using PAMAP2 dataset. That’s why we chose Random Forests for our work.

In our paper, we use Random Forest classification algorithm to achieve accurate human activity recognition while conserving the sensor nodes lifetime. After that, we exploit the result of this activity recognition to make routing in WBANs more efficient. By tracking the user activity and movement, we dynamically adjust routing decisions based on the recognized activity. This approach improves data transmission, making WBANs more efficient and reliable. Recently, there are a lot of researches that exploit machine learning algorithms in improving the routing of WBAN but for best of our knowledge the utilization of HAR in WBAN routing has not been discussed before. Which is considered the main contribution of our work.

Now, we will discuss some of ML algorithms that is used to perform human activity recognition^31^ proposed TCN-attention-HAR which integrates Temporal Convolutional Networks (TCNs) with an attention mechanism time convolutional network to enhance human activity recognition (HAR) by the efficient detection of both temporal and spatial dependencies in multivariate time-series data. The model utilize TCNs’ capability to process long-term dependencies through expanded convolutions, while the attention mechanism improves the focus on related time steps and sensor features. This model is applied to various datasets like PAMAP2 which is the data set used in our work, TCN-attention-HAR which prove superiority performance in contract to conventional models like CNNs and RNNs, achieving higher accuracy, precision, and F1-scores. Compared to our work, we use sliding window approach which reduce dataset size while preserving all 54 features reinforced with extra statistical metrics which carry useful and relevant information of diverse aspects of user activities that help Random forest model to recognize the activity of person in all cases, which can help the model better differentiate between classes or predict outputs accurately. This what make our model outperform these traditional multi-channel CNN attention methods by approximately 1.13%, 1.83% and 0.51%, respectively on available datasets WISDM, Pamap2, and USC-HAD. Additionally we exploit the trained model to enhance routing of WBAN by choosing the best path based on recognized activity. Our model achieves accuracy close to TCN-attention-HAR but at a lower computation cost which make our model optimal for resource constrained WBAN. Comparing computation cost of our model and TCN-attention-HAR we find that the computational costs of TCN-attention-HAR is combination of its two primary components: the Temporal Convolutional Network (TCN) and the Attention Mechanism. The complexity of TCN is O(L.N.F.K) where L is the number of layers in the TCN, N is the Length of the input sequence, F is the Number of filters (output channels) in each convolution, K is Kernel size (window size for each convolution). The complexity of attention mechanism $O(H.N. d^{2})$ Where d be the feature dimension, N input steps, H is the number of attention heads. The total complexity of TCN-Attention-HAR: $O(L.N.F.K+H.N.d^{2})$ which is considered high compared to random forest complexity which prove the superiority of our model in energy constrained WBAN.

^32^ propose the integration of edge computing with a Bidirectional Long Short-Term Memory (BiLSTM) model for human activity recognition (HAR) using PAMAP2 dataset. In this model the data is processed locally on edge devices, which reduces delay and communication overhead, to recognize activity in real-time. This model faces challenges such as constrained computational resources on edge devices, the need for complex model optimization, deployment concerns in different real-world scenarios, and potential energy consumption concerns which can consume the batteries of WBAN sensor nodes more quickly unlike our model which conserves the WBAN resources. Table 1 shows that the computation cost of the proposed random forest algorithm model has lowest complexity than LSTM and other models.

The study in^33^ demonstrates the development of self-supervised learning for human activity recognition in wearable data, introducing a scalable and efficient solution for making use of unlabeled data. It highlights how Self-Supervised Learning framework can reduce the dependency on labeled data, improve representation learning, and enhance model robustness across diverse applications in health monitoring, fitness tracking. However, computational demands and data quality remain challenges for practical implementation.

The study in^34^ introduces the HARCNN model, a deep learning model that is designed specifically for Human Activity Recognition. This model involves ten convolutional blocks, each integrating convolutional layers, ReLU activation functions, and batch normalization layers. The output of the Specific blocks is merged with the help of depth concatenation, and max-pooling layers to decrease spatial dimensions. The model was evaluated on different datasets UCI-HAR, KU-HAR, WIDWM, and HMDB51 which achieve high accuracy and robustness across different temporal window sizes. This work focused dependently on activity recognition without utilizing its application in routing or data communication. Our model couples HAR with WBAN routing, using recognized activities to adapt routing paths dynamically, enhancing energy efficiency and network performance.

The study in^35^ proposes a simple, ontology-based approach to human activity recognition (HAR) that utilizes multisource data from public wearables like watches, shoes, and smart glasses. Rather than depending on complex machine learning models, it makes use of logical reasoning by using OWL2 ontologies for the classification of daily activities which depends on the motion of sensor data, fulfilling over 90% accuracy among various scenarios with minimum training data. This method concentrated on activity recognition based on individual characteristics without taking into consideration routing decisions and network-level optimizations. This indicates the novelty of our work in utilizing HAR in WBAN real-time data routing which makes it more intelligent and improves the performance of the WBAN network.

The study in^36^ presents an adaptive Reinforcement Learning -based routing protocol that improve the performance of heterogeneous WBANs by taking into consideration mobility and energy efficiency challenges. A reinforcement learning algorithm trains the WBAN to choose the optimal path based on factors such as link quality, energy consumption, and mobility patterns. Our model choose the routing path based on recognized activity which can solve the disconnection resulted in mobility and topological change which has not been addressed before.

Currently, research efforts are either focusing on employing activity recognition using various machine learning algorithms to improve the performance of health care system using WBANs such as for human fall detection or applying machine learning algorithms directly to improve performance of WBAN such as improving the routing protocols^37–40^. However, little attention has been given to integrating activity recognition into the routing protocols to dynamically adjust routing decisions based on the recognized activity. This work addresses this gap by using activity recognition into the routing process. Additionally, we employ the random forest protocol, which has demonstrated suitability for the WBAN environment due to its low energy requirements. This approach achieves significant improvements in data transmission efficiency and prolongs network lifetime compared to other recent studies.

**Table 1:** Comparison of training and prediction complexities for machine learning models.

Model	Training complexity	Prediction complexity
Random forest	$O(T \cdot F \cdot N \cdot D)$	$O(T \cdot D)$
Support vector machines (SVM)	$O(N^2 \cdot M)$ (linear); $O(N^3)$ (non-linear)	O(S.M)
Hidden Markov models (HMM)	$O(I \cdot N \cdot S^2)$	$O(N \cdot S^2)$
Decision trees	$O(N \cdot M \cdot D)$	*O*(*D*).
Recurrent neural networks (RNN)	$O(E \cdot N \cdot T \cdot H^2)$	$O(T \cdot H^2)$
Temporal convolutional networks (TCN)	$O(E \cdot N \cdot F \cdot K)$	$O(T \cdot K)$
TCN-Attention-HAR	$O(E \cdot N \cdot H^2)$	$O(T \cdot H^2)$
Convolutional neural networks (CNN)	$O(E \cdot N \cdot F \cdot K^2)$	$O(F \cdot K^2)$
Reinforcement learning	$O(S \cdot A \cdot T \cdot E)$	$O(S \cdot A)$
LSTM	$O(E \cdot N \cdot T \cdot H^2)$	$O(T \cdot H^2)$

Where

N = Number of samples

T = Number of trees

F = Number of features

D = Depth of the tree

M = Number of classes or categories

S = Number of states (HMM), number of support vectors (SVM)

E = Epochs

H = Number of hidden units (RNN)

A = Number of actions (Reinforcement Learning)

I = Iterations for training (HMM)

T = Sequence length or time steps

## Physical activity recognition model

Physical activity recognition is an important area of research, with applications in health, wellness, fitness, and sports. Wearable sensors such as accelerometers and gyroscopes can be used to collect data on human movement, which can then be analyzed using machine learning algorithms to predict different types of physical activities which in turn has a great effect on the selection of the optimal path to route data. In our model the PAMAP2 dataset is trained using random forest classification algorithm which is considered the least power consumption algorithm so it is the best suited algorithm to be used in WBAN as it requires minimum power consumption to conserve the energy of its sensor nodes.

## PAMAP2 dataset description

The PAMAP2 dataset is a publicly available dataset of physical activity monitoring that was collected by the Pervasive Systems Group at ETH Zurich in Switzerland. The dataset consists of 18 different activities, including walking, jogging, cycling, rowing, and various household and lifestyle activities. The activities were performed in two different sessions, separated by several weeks, to capture variations in the participants’ behavior over time.

The sensor data was collected at a sampling rate of 100 Hz and preprocessed to remove gravity components and apply noise filtering. The dataset also includes annotations for each activity segment, indicating the start and end times of each activity.

The dataset is provided in the form of text files, with one file per participant and session. Each file contains multiple columns of sensor data, including accelerometer, gyroscope, and magnetometer readings, as well as annotations for each activity segment.

## Model training and evaluation

In our training process, we take specific steps to prepare the dataset for machine learning. First, we clean the data to fix any inconsistencies or anomalies in the data. Then, we preprocess the data to make sure it’s in the right format for analysis. After that, we normalize the accelerometer and gyroscope data so that they have a zero mean and unit variance, making sure that the features are comparable and not influenced by differences in sensor readings or participants. Then We separate labels (Feature Selection).

In our model the selected features of the PAMAP2 dataset, are all columns except the last one, which contains the activity labels. These extracted features (sensor readings) is considered the input to the model, and the activity labels is the target variable. Next, we apply Sliding Window for feature extraction of PAMAP2 dataset as follows:

First we will define Window Parameters which is:

Window Size (W): 5 seconds at sampling rate 100 Hz (W=5$\times$100=500 rows per window for each sensor) and Step Size(S): 2 seconds. For 100 Hz, S=2$\times$100=200 rows per step). This means that the window (500 samples) will shift with a step of 2 seconds, therefore, we’ll extract one window at a time, slide it by 2 seconds, and repeat the process until the all dataset is covered. i.e the first Window will has data from 0 to 5 seconds then it will shifted by 2 seconds, so the second Window will has data from 2 to 7 seconds and so on.

As $W=500$ rows per window, $S=200$ rows per step then the overlap is $500-200=300$ rows of overlap between successive windows. This overlap shows that each new window shares some of its time steps with the previous window. This overlap helps the model ensures continuity in the activity patterns and prevents sudden changes between sequences.

Apply Windowing for each activity standing, lying,...etc. will aid in reducing the number of rows in all dataset which in turn decrease the computation cost.

Now, we will extract relevant statistical features(standard deviation, mean, median, min, max, range) for each of the 54 features(from the data of each window for each sensor). For all 54 features, the dataset will have 54$\times$6=324 aggregated features per window. The selected statistical features (standard deviation, mean, median, minimum, maximum, and range) were chosen due to their capability to summarize the characteristics of the raw sensor data across time windows successfully. We consider these features as relevant because they catch important patterns and variations in the data, such as central tendency (mean, median), variability (standard deviation), and extremes (min, max, range). These attributes provide a comprehensive representation of sensor behavior during different activities. For example, the mean and median show overall activity directions, while the range and standard deviation show fluctuations, aiding in the differentiation of dynamic activities (ex. running) from static ones (ex. sitting). Aggregating these features across all 54 sensors ensures strong and detailed dataset with 324 features per window, optimizing the model’s capacity to accurately classify activities while maintaining computational efficiency. Finally, we split the dataset into training and testing sets in an 80:20 ratio to properly evaluate and assess the model’s performance. These steps are essential for making sure our machine learning algorithm are reliable and effective.

**Fig. 1:**
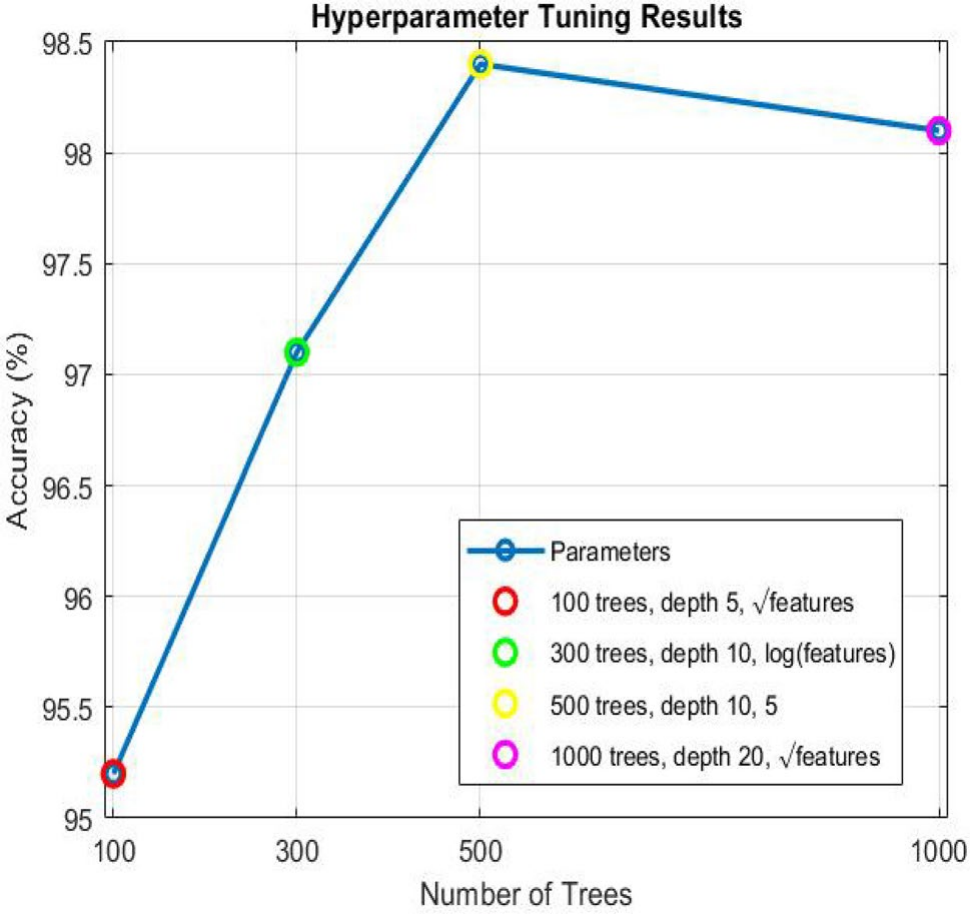
Comparison of accuracy between different parameters.

After these aforementioned preprocessing steps, random forest classification algorithm is trained on the selected features using PAMAP2 training dataset. The hyperparameters of the random forest, such as the number of trees, the depth of each tree, and the number of features considered at each split, were optimized using cross-validation techniques. To choose the best performing hyperparameters that give the most accurate results, we used Grid Search for hyperparameters tunning of our random forest algorithm. Grid Search typically tries different combinations of hyperparameters, such as max depth, number of trees, and max features. For each combination, cross-validation is applied to evaluate the performance of the model on different groups of the data. The combination of hyperparameters that achieves the highest average cross-validation result is then chosen as the best hyperparameters. After that we train the random forest algorithm with varying hyperparameters on PAMAP2 dataset and then evaluate the accuracy of these hyperparameters. The training result of our model using different parameters is shown in Table 2 and explained in Fig. 1.

**Table 2:** Performance Metrics by each class.

Hyperparameter set	No of trees	Max depth	Features at each split	Accuracy (%)
parameters 1	100	5	$\sqrt{features}$	95.2
parameters 2	300	10	$\log (features)$	97.1
parameters 3	500	10	5	98.4
parameters 4	1000	20	$\sqrt{features}$	98.1

As can be seen from Table 2

- test set parameters 1 (100 trees, depth 5, $\sqrt{features}$) shows the lowest accuracy at 95.2% as fewer trees and a shallow depth might not capture the complexity of the data well.
- test set parameters 2 (300 trees, depth 10, log(features)) improves accuracy to 97.1%, indicating that increasing the number of trees and depth has a positive impact.
- test set parameters 3 (500 trees, depth 10, 5 features) achieves the highest accuracy of 98.4%, shows the best-performing results.
- test set parameters 4 (1000 trees, depth 20, $\sqrt{features}$) slightly decreases accuracy to 98.1%, as beyond a certain point, increasing the number of trees decreases accuracy, as the additional trees may cause overfitting.Therefore, we choose the best-performing set was with 500 trees, a depth of 10, and 5 features at each split, resulting in 98.4% accuracy.

Where: Number of trees is used to form the random forest algorithm where each tree in the algorithm is trained on a different random subset of the data and features. As the number of trees increases the model become more accurate and stable, maximum depth of each tree determines how deep the tree can split into nodes. The deeper the tree, the more splits there are, which can lead to more specific and detailed decisions, features at each split parameter determines how many features (or columns) that the random forest algorithm can use at each split in a tree. PAMAP2 dataset contain 54 feature. For each split the random forest algorithm randomly choose for example n features. In the next split choose another random n features and so on. In our model, the best max feature = 5 which ensure the randomness into the model, that prevent overfitting by make sure that each tree in the Random Forest does not depend on the same features, thus ensuring diversity which leads to a more accurate model.

Using test parameters 3 the performance of the trained random forest classifier was evaluated using the following metrics:

1. Accuracy: The overall correctness of the model.
  1 $$\begin{aligned} {\text{Accuracy}}\,=\,\frac{TP+TN}{TP+FB+TN+FN+TN} \end{aligned}$$
2. Precision: The proportion of positive identifications that were actually correct.
  2 $$\begin{aligned} {\text{Precision}}\,=\,\frac{TP}{TP+FB} \end{aligned}$$
3. Recall (Sensitivity or True Positive Rate): The proportion of actual positives that were correctly identified.
  3 $$\begin{aligned} {\text{Accuracy}}\,=\,\frac{TP}{TP+FN} \end{aligned}$$
4. F1 Score: The harmonic mean of precision and recall, providing a balance between the two.
  4 $$F1{\mkern 1mu} {\text{Score}}{\mkern 1mu} = {\mkern 1mu} \frac{{2*{\text{Precision}}*{\text{Recall}}}}{{{\text{Precision}} + {\text{Recall}}}}$$
  Where:
  True positive (TP): The number of instances that were correctly predicted as positive
  False positive (FP): The number of instances that were incorrectly predicted as positive
  True negative (TN): The number of instances that were correctly predicted as negative
  False negative (FN): The number of instances that were incorrectly predicted as negative.
5. Confusion matrix: The confusion matrix represents the performance of our classification model that predicts the different activities of PAMAP2 dataset (e.g., lying, sitting, standing, walking, etc.). The rows correspond to the actual activities, and the columns correspond to the predicted activities. The diagonal elements show the number of correct predictions for each activity, while the off-diagonal elements represent misclassifications. This matrix shown in Table 3 shows that our model achieve high accuracy where most diagonal elements have high values, indicating that the model correctly predicts the activities most of the time. Additionally, it shows that the misclassifications are low, but noticeable. for detailed explanation of our classification model we illustrate the performance of each class in terms of Precision, Recall and F1-Score as shown in Table 4 then calculate their average as shown in Table 5.

**Table 3:** Confusion matrix of our proposed model.

	Lying	Sitting	Stand	Walk	Run	Cycling	Nordic walk	Ascending	Descend	Norm walk	Vacuum	Ironing	Rope
Lying	780	2	3	1	1	0	1	1	2	2	1	0	0
Sitting	2	499	1	2	1	0	2	1	1	3	2	1	0
Standing	2	1	500	2	3	0	1	1	0	1	0	1	0
Walking	2	1	2	800	5	1	4	2	1	3	0	0	1
Running	2	1	2	6	854	1	3	2	2	5	0	0	0
Cycling	0	0	0	1	0	765	1	2	3	1	0	0	0
Nordic Walk	0	0	1	4	2	1	633	2	1	2	0	0	1
Ascend	0	0	1	3	2	5	3	586	10	5	0	0	0
Descend	3	0	0	1	0	4	1	9	622	2	0	1	0
Norm walk	4	3	5	8	6	3	8	3	2	795	0	1	0
Vacuum	1	1	5	0	1	2	4	0	0	0	769	20	0
Ironing	0	2	1	0	0	0	0	0	0	1	4	532	0
Rope	0	0	0	0	1	1	0	3	1	1	0	1	230

**Table 4:** The performance of each class.

Class	Accuracy	Precision	Recall	F1-Score
Lying	0.982	0.992	0.992	0.992
Sitting	0.964	0.978	0.984	0.981
Standing	0.985	0.990	0.980	0.985
Walking	0.972	0.978	0.988	0.983
Running	0.973	0.980	0.982	0.981
Cycling	0.972	0.992	0.993	0.993
Nordic Walk	0.973	0.990	0.996	0.993
Ascend	0.958	0.954	0.971	0.962
Descend	0.972	0.979	0.978	0.978
Norm Walk	0.968	0.982	0.981	0.981
Vacuum	0.974	0.973	0.996	0.984
Ironing	0.967	0.994	0.960	0.977
Rope	0.973	0.987	0.999	0.990

**Table 5:** Average performance metrics across all classes.

Metric	Value (%)
Accuracy	98.6
Average precision	98.86
Average recall	98.68
Average F1-score	98.77

As mentioned above tunning the random forest parameters has a great impact on algorithm performance and accuracy.So we tune our random forest parameters so that it acheive the best performance result. Now we will prove that our choice of random forest with the above tuned parameters is the optimal solution for our WBAN application.

### Model validation details

To ensure the reliability of our model, we performed a simple validation model using our PAMAP2 Dataset, we choose PAMAP2 Dataset because of to its variety of sensor data which is considered a good choice for accurate activity recognition.

First we divided our dataset into 80% training and 20% testing. After that we perform the validation from the training data (from the 80% training data, we take 10% for validation and the rest (70%) for actual training.

The 20% for testing was kept and used only for final testing.

We performed 5-fold cross-validation on the training data( 70% of the dataset) to check the consistency and stability of our model. For each iteration, the data was divided into five equal parts one part for validation and the remaining five parts for training. This procedure was iterated five times, each time with different validation fold.

After training, the model was validated on the 10% validation set with result showed in Table 6. The validation has a major role in model hyperparameter tunning and identifying possible overfitting before final testing.

For the classifier, we used Random Forest due to its high accuracy, robustness to overfitting, and most importantly, low computational footprint, which aligns with the power constraints in WBAN devices.

**Table 6:** Validation Results of each fold.

Fold	Accuracy (%)
1	98.52
2	98.46
3	98.61
4	98.55
5	98.63

mean Accuracy 98.4 % Standard Deviation: 0.188 % The small standard deviation indicates that the performance of our model is consistent across different test sets.

Validation helps strengthen the reliability of the model by verifying its performance on an additional set before final testing.

## Analysis and comparison of results and complexity between our model and other HAR Models on PAMAP2 Dataset

In this section we will prove that our model is the most appropriate algorithm used for activity recognition in low power WBAN using PAMAP2 dataset.

First, we will compute the computation complexity of our model when applying a sliding window approach and extracting statistical features compared to various models on the same dataset (PAMAP2) with same features and parameters to ensure fair comparison.

Parameters of for PAMAP2 Dataset and sliding window approach

- Sampling rate: 100 Hz.
- Window size: $W = 500$ rows (500 samples per window).
- Step size: $S = 200$ rows (200 samples per step).
- Number of sensors (features): $M = 54$.
- Statistical features: $F = 6$ (mean, median, std, max, min, range).
- Total dataset samples: $N = 29,160,000$.

### Number of windows

The number of windows *K* is calculated as:

5 $$\begin{aligned} K = \frac{N - W}{S} + 1 \end{aligned}$$

To compute the computation complexity of our model we need to compute the feature extraction cost

### Computational cost for feature extraction

For each window:

- Compute statistical features (mean, median, std, max, min, range) for each of the 54 features. Each statistical operation over the window of size *W* has a complexity of *O*(*W*).
- Total operations per feature: $O(F \cdot W)$, where $F = 6$.
- Total for all 54 features: $O(M \cdot F \cdot W)$, where $M = 54$.

For *K* windows, the total cost is:

6 $$\begin{aligned} O(K \cdot M \cdot F \cdot W) \end{aligned}$$

Substituting $$K = \frac{N - W}{S} + 1$$:

7 $$\begin{aligned} O\left( \left( \frac{N - W}{S} + 1\right) \cdot M \cdot F \cdot W\right) \end{aligned}$$

Simplify:

8 $$\begin{aligned} O\left( \frac{N \cdot M \cdot F \cdot W}{S}\right) \end{aligned}$$

Given

- Sampling rate: 100 Hz.
- Window size: $W = 500$ rows.
- Step size: $S = 200$ rows.
- Number of features: $M = 54$.
- Statistical features: $$F = 6$$.
- Total dataset samples: *N* = 29,160,000.

Substitute values:

9 $$\begin{aligned} O\left( \frac{N \cdot 54 \cdot 6 \cdot 500}{200}\right) = O(405 \cdot N) \end{aligned}$$

According to PAMAP2 dataset the recording duration of each participant equals approximately 10 hours.

PAMAP2 dataset consists of 9 participants and is considered continuous, then, the total samples are:

N=sampling rate $\times$ recording duration in seconds $\times$ number of participants (for 10 hours per participant)

Samples per participant=100 $\times$ 10 $\times$ 3600 =3,6000,00 samples per participant.

total samples N= 3600,6000,00 $$\times$$ 9=32,400,000

Total Number of Windows (K)

From previous sliding window setup:

- Window size (W): 500 rows.
- Step size (S): 200 rows.

The formula for the total number of windows is:

$$K = N - \frac{W}{S} + 1$$

Substituting $$N = 32,400,000$$:

$$K = 32,400,000 - \frac{500,200}{200} + 1$$

Calculating:

$$K = \frac{32,400,000 - 500}{200} + 1 \approx 145,797.5 + 1 \approx 161,998 \text { windows.}$$

### Computation per window

For each window:

- $$M = 54$$ features,
- $F = 6$ statistical features per sensor.

Total operations per window:

$$O(M \cdot F \cdot W) = 54 \cdot 6 \cdot 500 = 162,000 \text { operations.}$$

## Total computational cost

Total cost over all windows:

$$O(K \cdot M \cdot F \cdot W)$$

Substituting:

$$O(161,998 \cdot 54 \cdot 6 \cdot 500) = 26,243,67,000 \text { operations.}$$

For fair comparison between our Random Forest model(RF) and all previous models, we need to justify the parameters of other models so that it ensures equivalence in terms of size of dataset, representation of features and computational requirements(same feature extraction using same sliding window parameters ).

Sliding Window Preprocessing:

- K=161,998 total windows derived from sliding a window of 500 samples with a step of 200 samples.
- F=324: Aggregated statistical features extracted per window.

Random Forest Parameters:

- T=500: Number of trees.
- D=10: Maximum tree depth.

Recurrent Models (GRU, LSTM, etc.):

- H=128: Hidden units.
- T=500: Sequence length (5 seconds at 100 Hz sampling rate).
- E=10: Number of epochs for training.

CNN Parameters:

- K=3: Kernel size.

### Complexity of random forest

- Training complexity:
  $$\begin{aligned} ( O(T \cdot F \cdot K \cdot D)) \end{aligned}$$
  =500$$\times$$ 324$$\times$$ 161,998 $$\times$$ 10= 262.5 $$\times 10^9$$
- Prediction complexity: $$O(T \cdot D)$$=500 $$\times$$ 10=5000

#### Complexity of SVM (Linear Kernel)

- Training complexity: $$O(K^2 \cdot F)$$=$$(161,998)^2 \cdot 324=8.52\times 10^{12}$$
- Prediction complexity: $O(S \cdot F)$ (where $S$ is the number of support vectors, assumed $S \propto N$)=$\sqrt{161,998} \times 324=1.3 \times 10^5$

### Complexity of recurrent models (GRU, LSTM, BiLSTM, etc.)

- Training complexity: $$O(E \cdot K \cdot T \cdot H^2)$$=$$10 \times 161,998 \times (128)^2=2.6 \times 10^{13}$$
- Prediction complexity: $$O(T \cdot H^2)$$=$$500 \times (128)^2=8.19 ^6$$

### Bidirectional models (BiLSTM, BiGRU)

- Training complexity: $$O(E \cdot K \cdot T \cdot 2H^2)$$=$$10 \times 161,998 \times 500 \times 2(128)^2=2.38\times 10^{12}$$
- Prediction complexity: $$O(2T \cdot H^2)$$= $$2 \times 500 \times (128)^2=1.64 \times 10^7$$

### CNN-based models (e.g., CNN-GRU , CNN-LSTM)

- Training complexity: $$O(E \cdot K \cdot F \cdot Ker)$$=$$10 \times 161,998 \times 324 \times 3 =1.57 \times 10^{10}$$
- Prediction complexity: $$O(T \cdot H^2)$$= $$500 \times (128)^2= 8.19 ^6$$

**Table 7:** Complexity Comparison between our RF model and other models.

Model	Training Complexity ($O$)	Prediction Complexity ($O$)
Random Forest (RF)	$O(T \cdot F \cdot K \cdot D) = 262.5 \times 10^9$	$O(T \cdot D)= 5,000$
SVM (Linear Kernel)^19,20^	$O(K^2 \cdot F) = 8.52 \times 10^{12}$	$O(S \cdot F) = 1.3 \times 10^5$
GRU^31^	$O(E \cdot K \cdot T \cdot H^2) = 2.6 \times 10^{13}$	$O(T \cdot H^2) = 8.196 \times 10^6$
LSTM^32^	$O(E \cdot K \cdot T \cdot H^2) = 2.6 \times 10^{13}$	$O(T \cdot H^2) = 8.196 \times 10^6$
BiLSTM^32^	$O(E \cdot K \cdot T \cdot 2H^2) = 2.38 \times 10^{12}$	$O(2T \cdot H^2) = 1.64 \times 10^7$
CNN-GRU^41^	$O(E \cdot K \cdot F \cdot Ker) = 1.57 \times 10^{10}$	$O(T \cdot H^2) = 8.196 \times 10^6$
CNN-LSTM^42^	$O(E \cdot K \cdot F \cdot Ker) = 1.57 \times 10^{10}$	$O(T \cdot H^2) = 8.196 \times 10^6$
CNN-BiLSTM^43^	$O(E \cdot K \cdot F \cdot Ker) = 2 \cdot 1.57 \times 10^{10}$	$O(2T \cdot H^2) = 1.64 \times 10^7$
CNN-A-BiGRU^44^	$O(E \cdot K \cdot F \cdot Ker) = 2 \cdot 1.57 \times 10^{10}$	$O(2T \cdot H^2) = 1.64 \times 10^7$

**Table 8:** Comparison of Performance Metrics between our random forest model and Different Models.

Model	Precision	Recall	F1-Score	Accuracy
Random Forest	0.9886	0.9868	0.9877	0.986
SVM	0.8200	0.8941	0.8641	0.8033
HMM	0.9015	0.9453	0.9199	0.9019
Genetic Algorithm	0.9323	0.9556	0.9381	0.9345
GRU	0.7813	0.8461	0.8115	0.7905
GRU-Attention	0.9420	0.9581	0.9431	0.9412
CNN-GRU	0.8394	0.8963	0.8485	0.8409
LSTM	0.7663	0.8428	0.7955	0.7709
Attention-LSTM	0.7945	0.8628	0.8252	0.7994
BiLSTM	0.8570	0.8946	0.8644	0.8577
CNN-LSTM	0.8988	0.9347	0.9030	0.8983
CNN-BiLSTM	0.9102	0.9448	0.9062	0.9005
CNN-A-BiLSTM	0.9176	0.9414	0.9110	0.9130
CNN-BiGRU	0.8884	0.9264	0.8860	0.8831
TAHAR-Student-CNN	0.8987	0.8976	0.8482	0.8524
TAHAR-Student-LSTM	0.8948	0.9317	0.9079	0.8836
TAHAR-Student-GRU	0.8976	0.9317	0.8583	0.8701
TCN-Attention-HAR-Teacher	0.9423	0.9632	0.9488	0.9434

A can seen from Table 7 our random forest model is the least power consumption compared to other models especially during prediction and this is what concerns us, as the training of the model takes place outside the node, and what is done inside the node is the prediction. Now we will compare the results (Recall, Accuracy, Precision, F1-score) of our model compared to other models^31^ on the same PAMAP2 dataset

As can be seen from Table 8 that our random forest model outperform other models it is the most suitable for dataset and for the bank. because it the most suitable model for PAMAP2 dataset and forlow power WBAMs.

## Impact of human activity recognition on routing performance

Routing is the process of selecting path from source to destination. Routing is considered one of the most important performance metric of WBAN. However the choice of optimal path is challenging requirement addressed in this paper. The significance of routing efficiency lies in optimizing data transmission within resource-constrained WBANs, in the same time improving network performance and prolonging sensor node lifetimes. our paper achieve this trade off by making the routing more intelligent through the utilization of Human Activity Recognition (HAR) in routing decision. The proposed human activity recognition model using the PAMAP2 dataset can significantly enhance routing performance in WBANs. By detecting a user’s activity through our trained model, the routing protocol can adapt to current conditions, like signal strength and channel quality, to choose the best paths for routing. This helps ensure reliable communication by avoiding areas with weak connectivity and finding better routes.

During low-activity periods (like resting), fewer resources are allocated to conserve energy. On the other hand, when the user is active and needs continuous data transmission, more resources are allocated to maintain a stable connection. This approach also enhance energy-efficient routing by choosing paths that consume less power during low activity, which helps conserve battery life and optimize energy usage in the WBAN.

Additionally, the system can prioritize critical data during activities that require real-time monitoring or during emergency cases, ensuring fast and reliable data transfer. This is essential for time-sensitive applications and improves the overall quality of service (QoS) in the WBAN. Finally, we evaluate the performance of the integrated routing protocol with activity recognition. Assessing the impact of utilizing activity recognition on the routing decisions. We use metrics to measure the performance of the routing protocol, such as network lifetime, number of dead nodes, total remaining energy, and throughput. Iterating and optimizing the integration as needed to improve the overall performance and efficiency of the routing protocol in response to different activities.

## System model

WBAN consists of one sink node typically positioned around the waist or wrist and a set of N sensor nodes located at different places of the body to detect different signs. All sensor nodes transmit its sensed data to the sink node.

**Fig. 2:**
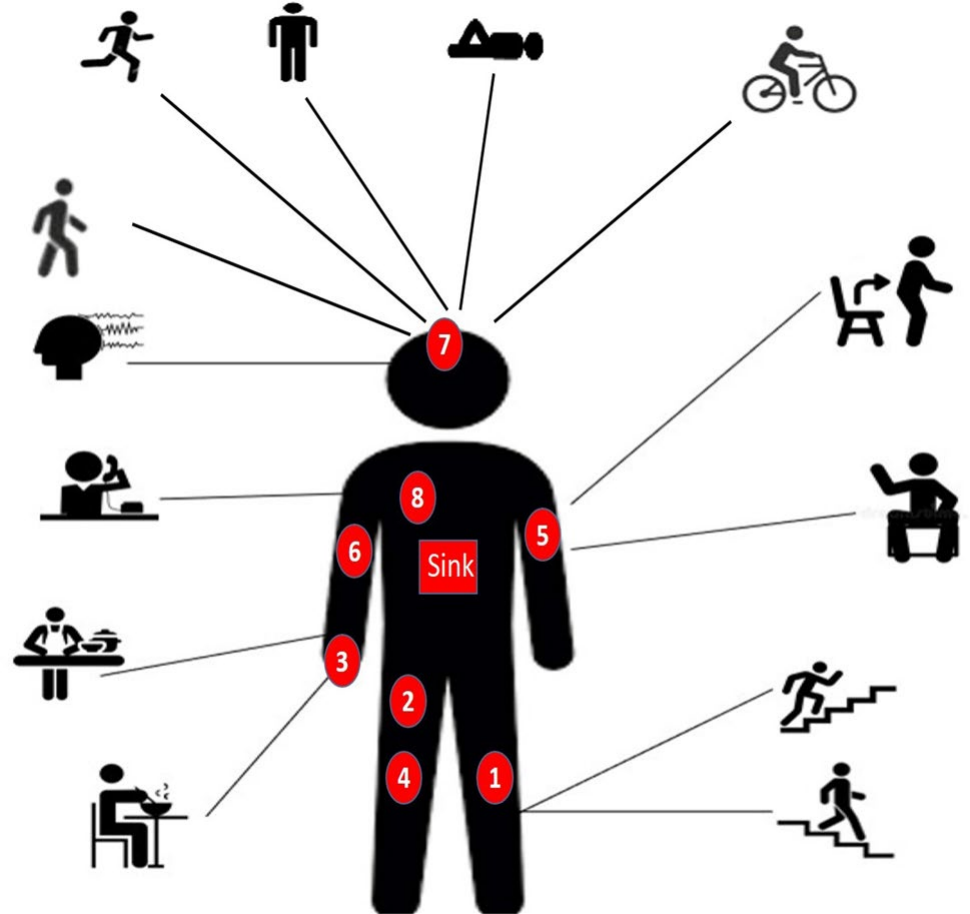
System model of our proposed protocol.

The user can perform different activities such as walking, running, cycling, nordic walking, rope jumping, sitting, standing, lying down, ascending stairs, descending stairs

## Proposed model

After training the activity recognition model, it is deployed to the sink node. The model takes all sensor nodes data as input, processes it to identify the user’s activity, and then makes routing decisions based on the recognized activity.

In our activity recognition model which shown in Fig. 2, the activities is classified into four types Resting, Walking, Exercising, and Emergency, based on this classification The routing procedure is as follows

1. Resting Activity: During the “Resting” activity, which typically includes sitting or lying down, the collected data remains relatively stable. Therefore, our routing protocol aims to conserve energy by transmitting the data every 15 minutes to minimize power consumption. The routing procedure in this case is multihop routing with minimal hop counts for more energy conservation.
2. Walking Activity: When the user transitions to “Walking,” indicating light physical activity, data from motion sensor nodes and heart rate monitors are transmitted more frequently (every 5 minutes) to monitor changes during physical activity. The routing procedure in this case selects routing paths with low latency and high data throughput to provide timely feedback to healthcare providers
3. Exercising Activity: During more intense “Exercising” activities like jogging or cycling, the routing protocol shifts towards real-time monitoring and prioritizes the transmission of vital signs data at a higher rate ( every 1 minute) to capture rapid physiological changes. The routing procedure in this case is direct data transmission to ensure periodic monitoring with low latency and high data throughput.
4. Activity Transition: When switching between activities, like moving from “Resting” to “Exercising,” the routing protocol adjusts gradually. It begins with energy-saving routes and slowly switches to faster, low-latency routes as the system confirms the change in activity. This smooth transition helps avoid sudden increases in data transmission or energy use.
5. Emergency Scenario: In case the activity recognition system detects an emergency case, such as a fall or abnormal heart rate during resting, walking or exercise activity, the routing protocol can override previous routing decisions. It can immediately establish a high-priority, low-latency path to transmit emergency data to the medical server.

Our work presents a novel framework that strongly integrates Human Activity Recognition (HAR) into the routing decisions of Wireless Body Area Networks (WBANs). As per our best knowledge, we didn’t find a publication that utilized activity recognition as an integral part of developing an adoptable WBAN routing mechanism. On the other hand, previous research has mainly focused on HAR or routing independently, our proposed model efficiently utilized the output of HAR to directly affect routing decisions in real-time based on the human activity criticality, current sensor energy, and overall network performance optimization. You can introduce a new figure showing that the ML model is using the HAR data to recognize the activity type, hence adjusting the routing mechanism based on the activity.

**Fig. 3:**
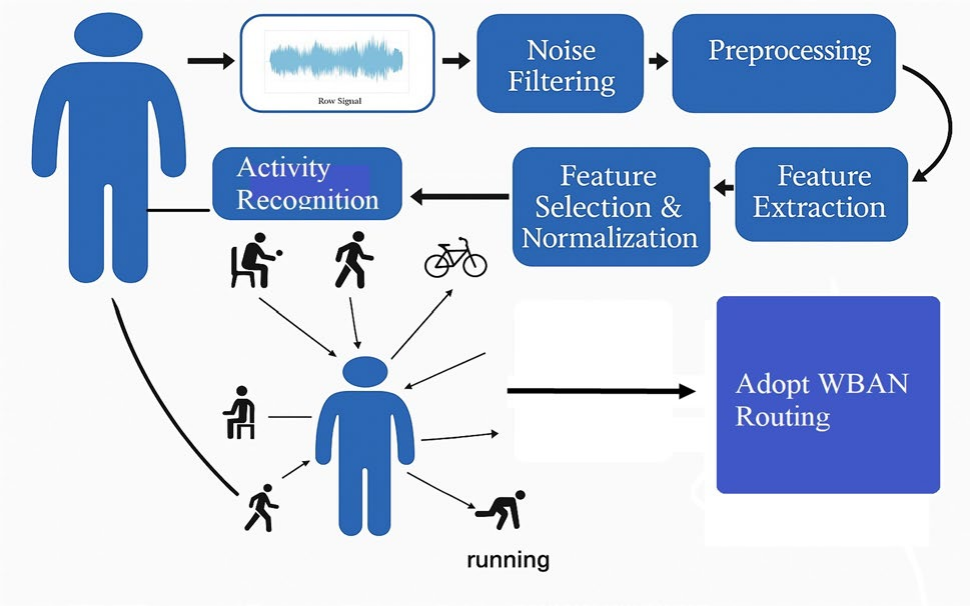
WBAN Routing based on HAR.

As shown in Fig. 3 the ML model is using the HAR data to recognize the activity type, hence adjusting the routing mechanism based on the activity.

The main contributions and novelties of our work are as follows: Integration between HAR and routing: different from existing works where HAR and routing are handled separately, our work integrates Random Forest-based HAR directly with WBAN routing procedures. Real-Time routing decision making: our model adjusts routing paths dynamically based on recognized user activities (e.g., walking, running, lying), which helps in efficient data transmission which in turn improves the overall WBAN performance. Energy-Efficient Communication: By adjusting routes based on user activity, our model reduces unwanted communication overhead and energy consumption. The choice of the PAMAP2 Dataset due to its variety of sensor data which is considered a good choice for accurate activity recognition.

### Algorithm: wireless body area network (WBAN) routing

**Figure:**
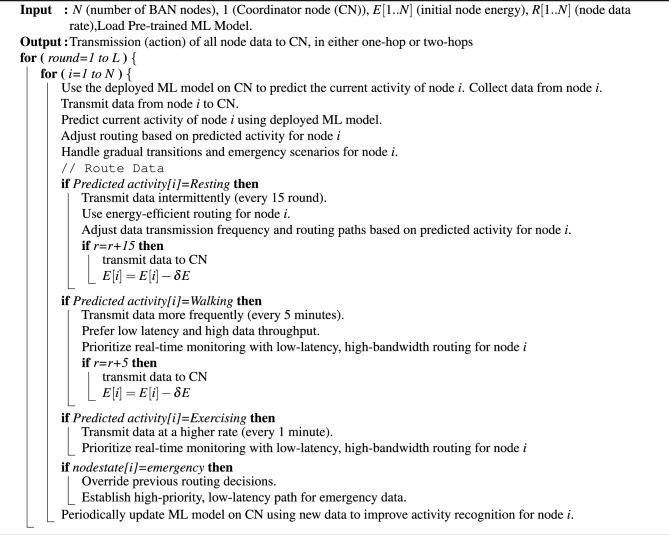
Algorithm 1 Proposed protocol

## Simulation results

To validate the effectiveness of our proposed protocol, we conducted simulations using Matlab. We compared our results with the published figures of mobTHE^45^, utilizing parameters outlined in Table 9. To ensure fairness in comparison, we maintained consistency with the parameters used in the aforementioned protocol. While the compared protocol typically run for 120000 rounds of simulation, we extended ours to 200000 rounds to highlight the performance enhancements of our proposed protocol over the others. We demonstrated our protocol’s superiority in metrics such as network lifetime, throughput, and overall residual energy.

The reasoning behind choosing mobTHE protocol in comparison to our protocol is that we apply our routing protocol on the same environment as mobTHE (mobile WBAN and same types and number of sensor nodes ). We consider various human activity and its impact on routing performance. Also mobTHE take into consideration the human mobility and its impact on routing but it didn’t make use of HAR. Therefore, the comparison clarify the impact of the utilization of HAR on routing performance and how it make our protocol more intelligent and improve the overall WBAN performance.

**Table 9:** Simulation parameters.

Parameter	Value
Number of simulation rounds, *L*	20000
Number of WBAN nodes, *n*	8
Initial node energy, $E_{i}, i \in [1..n]$	0.5 *J*
Packet size, *K*	4000
Path loss coefficient, pl	3.38
Node energy decrement per packet transmitted, $\delta E$	$6.68e^-4$+$2.6e^-4$*($D^{3.38}$)
Noise model	Additive white Gaussian noise (AWGN)

**Fig. 4:**
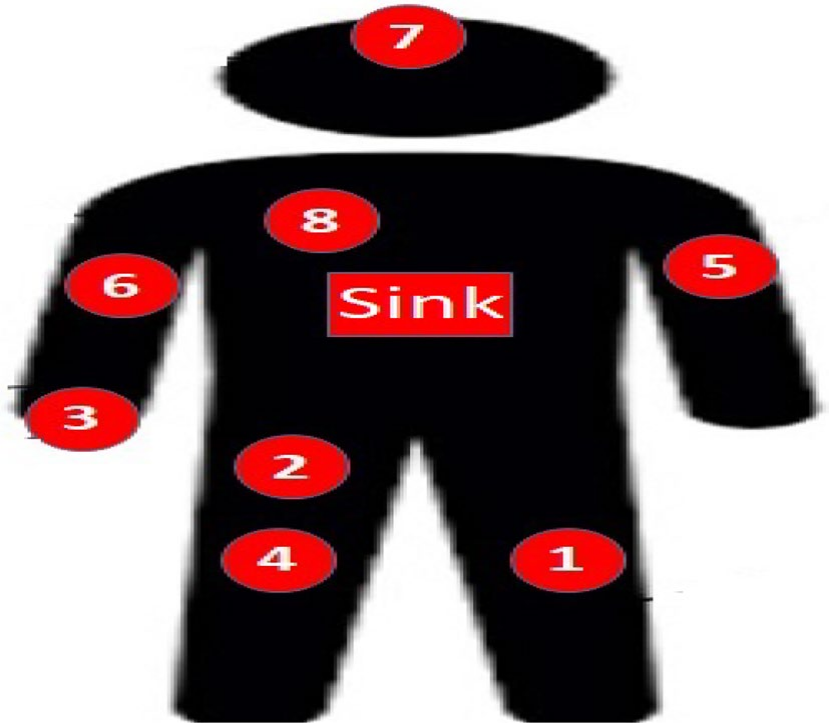
Deployment for the 8 nodes of the WBAN under the proposed protocol.

The 8 nodes of our WBAN have been added in their accurate place based on their function^46^ as shown in Fig. 4 are:

- Node 1, (temperature sensor), monitors body temperature.
- Node 2, (Insulin Pump), delivers insulin 24 hours a day.
- Node 3, SpO2 (peripheral capillary oxygen saturation), monitors the amount of oxygen in the blood.
- Node 4, EMG (Electromyography), monitors the electrical activity produced by skeletal muscles.
- Node 5, BP (Blood Presure) monitors blood pressure of the patient.
- Node 6, CGM (Continuous Glucose Monitoring), monitors glucose levels in real-time throughout the day and night to ensure the right amount of insulin is released at the right time.
- Node 7, EEG (Electroencephalography ), monitors electrical activity of the brain.
- Node 8, ECG (Electrocardiogram), monitors the electrical activity of the heart over a period of time.

We study the effect of applying random forest classification algorithm to recognize user activity on routing performance in terms of network lifetime, total remaining energy and throughput comparing results with mobTHE protocol. The training of the Random Forest model is performed outside the nodes to conserve their energy. We deploy only the trained results within the nodes. The training phase involves tunning hyperparameters to identify the optimal settings that give accurate results, not just in terms of model accuracy, but also based on the our performance metrics. We deploy the result of each parameter and test its result in terms of network lifetime, total remaining energy and throughput. We specifically choose metrics that help us achieve the most improvement in the overall WBAN performance

We use the best hyperparameters (500 trees, depth 10, 5 features) which achieve the highest accuracy of 98.4%

**Fig. 5:**
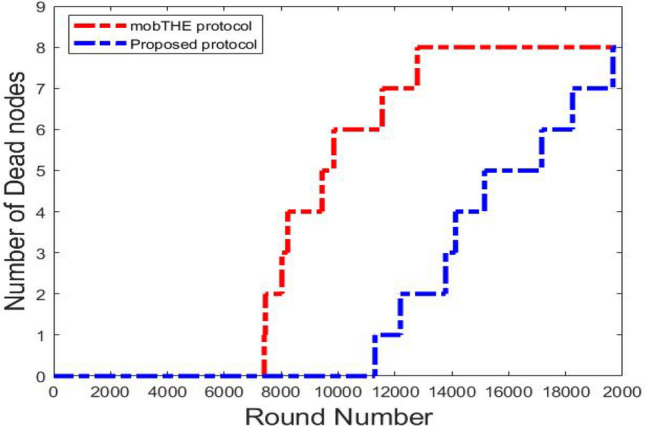
Number of dead nodes.

The degradation of WBAN lifetime can be observed through the number of dead nodes over time, represented in Fig. 5. This metric indicates the gradual reduction in the operational capability of the WBAN as nodes exhaust their energy resources. The network lifetime is the period from when the network starts working until it completely stops because the last node’s energy is exhausted Therefore, the stability of the WBAN refers to the time interval from the start of network operation until the dead of the first node.

As can be seen from the results, our protocol showed the first node failure at around round 11,800 and the last node failure at round 19,900. In comparison, the mobTHE protocol [42] had its first node failure at round 6,800, and the last node stopped at round 12,000. This shows that our protocol is better at saving node energy, extending the network’s life, and improving stability. This advantage comes from using a random forest classification algorithm to predict user activity and make better routing decisions. By classifying activities into “resting” and “exercising” states, and sending data less frequently (every 15 rounds) during resting periods, energy is saved, improving network stability and extending its overall lifetime. This energy-saving strategy is also shown in Fig. 5.

**Fig. 6:**
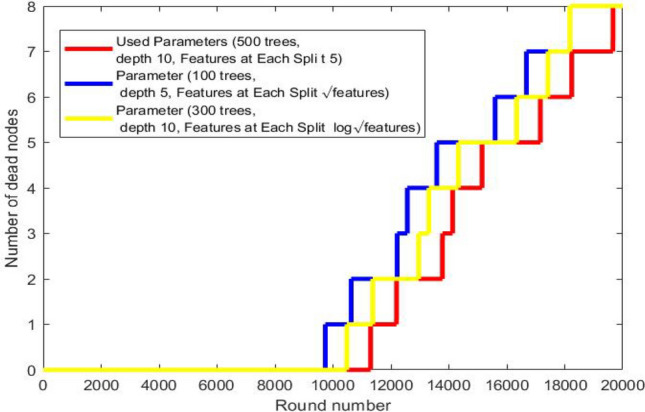
Number of dead nodes for each hyperparameter.

Fig. 6, compare the effect of different hyperparameters in the number of dead nodes.

As can be seen from the Fig. 6 the best hyperparameters (500 trees, depth 10, 5 features) extend the WBAN lifetime by approximately 2000 rounds more than the hyperparameters (300 trees, depth 10, Features at Each Split $log \sqrt{features}$) and by approximately 4000 round more than the hyperparameters (100 trees, depth 5, Features at Each Split features $\sqrt{features}$) which indicate the importance of tunning hyperparameters. The hyperparameter that gives the best result (accuracy), i.e. it makes the model predict the activity more accurately, which helps the routing protocol to take accurate routing decesion, which in turn conserve the node energy this leads to extending the network’s life.

**Fig. 7:**
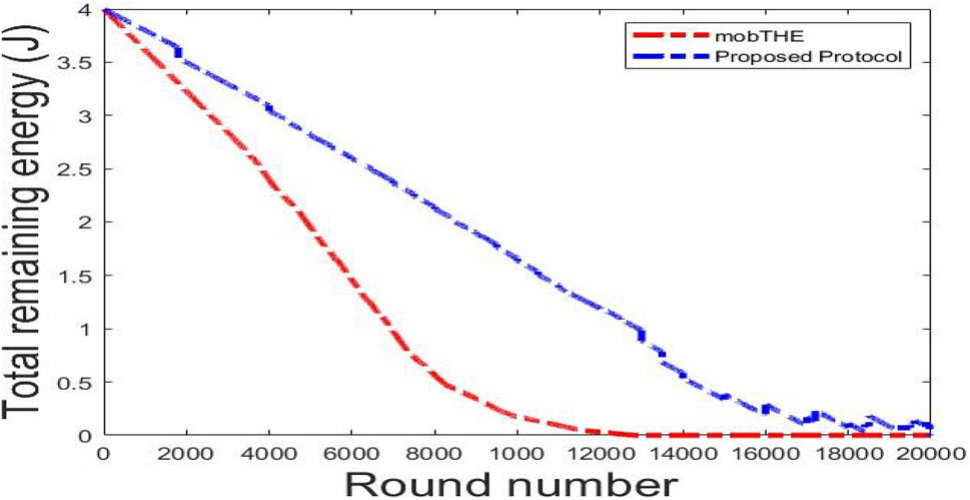
Total remaining energy.

**Fig. 8:**
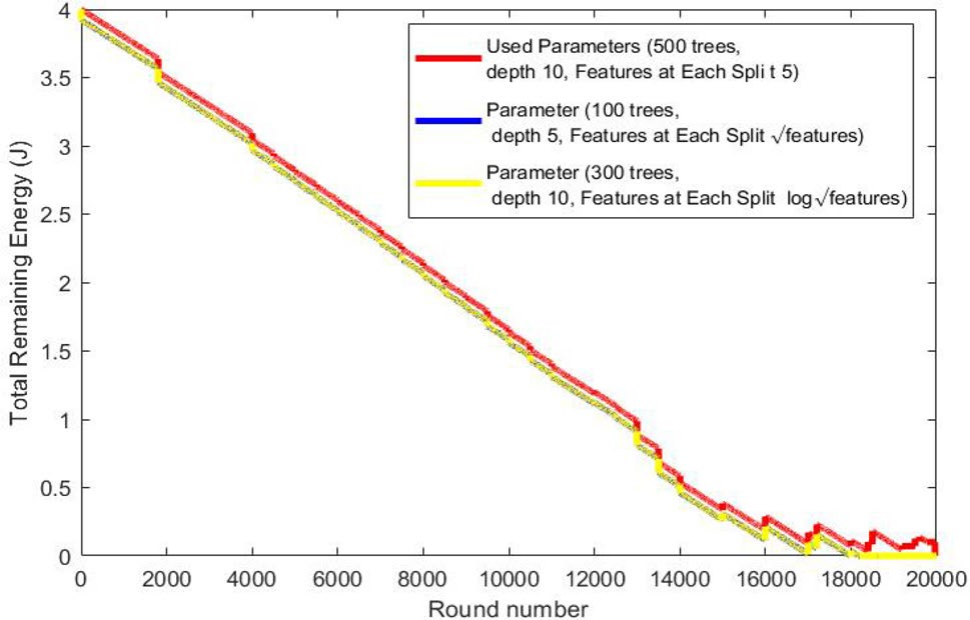
Total remaining energy for each hyperparameter.

**Fig. 9:**
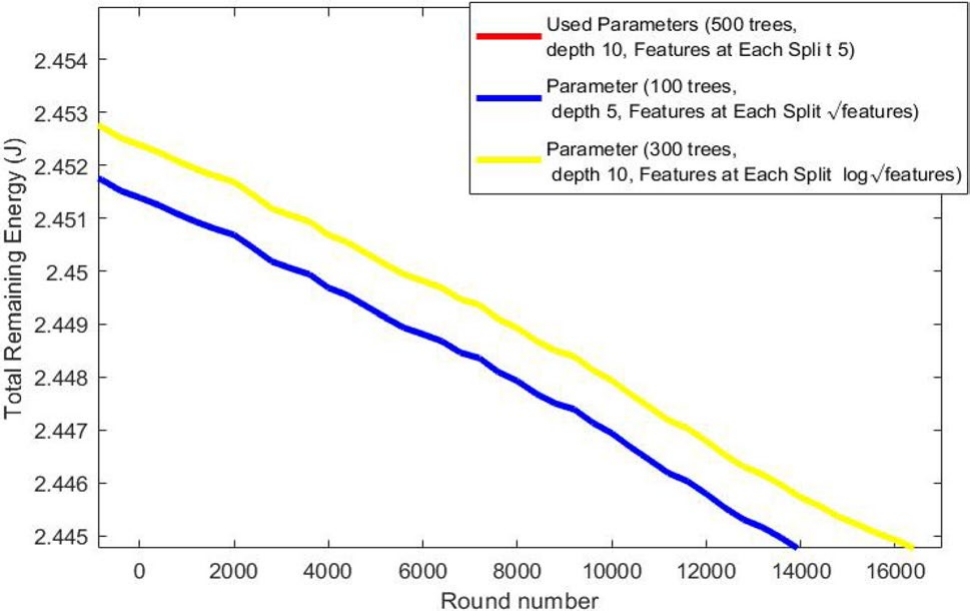
Total remaining energy for each hyperparameter.

Figure 7 shows how the total remaining energy in the network changes over time (represented by rounds ). It is clear that our protocol saves more energy than the mobTHE protocol. This energy saving comes from optimization of routing decisions based on recognizing user activities. When users are in a resting state, our protocol keeps nodes inactive and transmits data only every 15 rounds, which helps save energy and increases the total remaining energy. In contrast, the mobTHE protocol continuously transmits data, which deplets the node energy faster. This difference in routing decisions is what make our protocol outperform mobTHE protocol in terms of total remaining energy. Figures 8,9 illustrate the effect of different hyperparameters in the total remaining energy.

As can be seen from the Fig. 9 the best hyperparameters (500 trees, depth 10, 5 features) saves energy more than the hyperparameters (300 trees, depth 10, Features at Each Split $log \sqrt{features}$) and also more than the hyperparameters (100 trees, depth 5, Features at Each Split features $\sqrt{features}$) which prove the superiority of our hyperparameters utilized in the training that achieves accurate activity recognition which in turn make the routing protocol choose the best path that saves more energy.

**Fig. 10:**
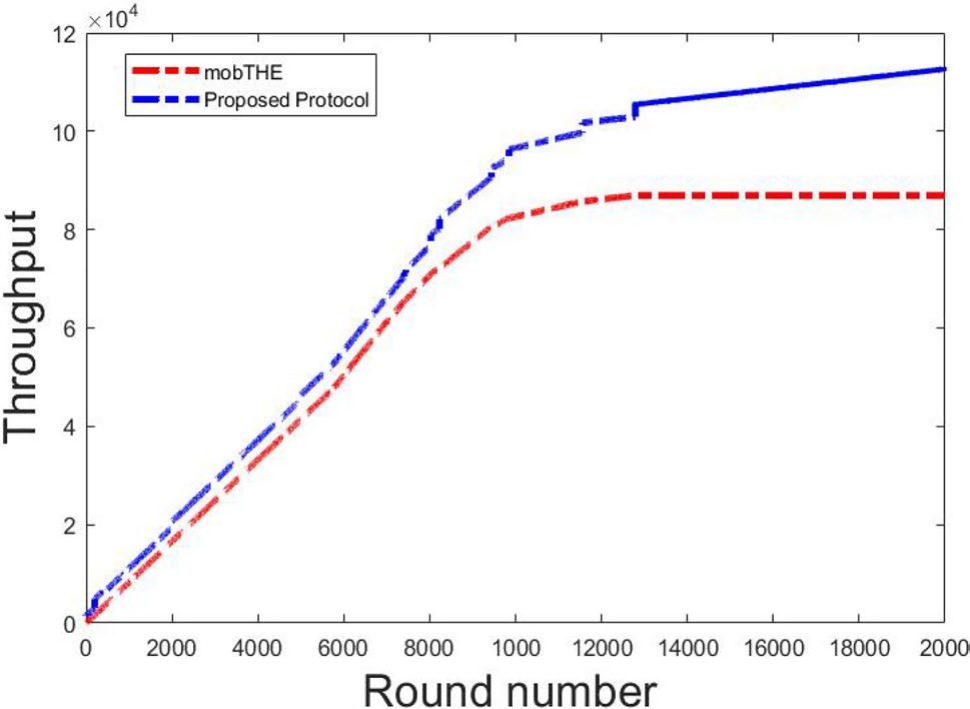
Throughput.

Figure 10 shows the throughput at each round. Our protocol achieves slightly better throughput than the mobTHE protocol, especially in the early rounds. This is because, in our protocol, nodes in a resting state send data every 15 rounds, reducing the total data sent to the sink node. However, the throughput slightly increasing but this improvement is small. Later on, as most nodes in the mobTHE protocol run out of energy and die, their throughput drops significantly this what make the throughput in our protocol outperforms mobTHE greatly in last rounds.

**Fig. 11:**
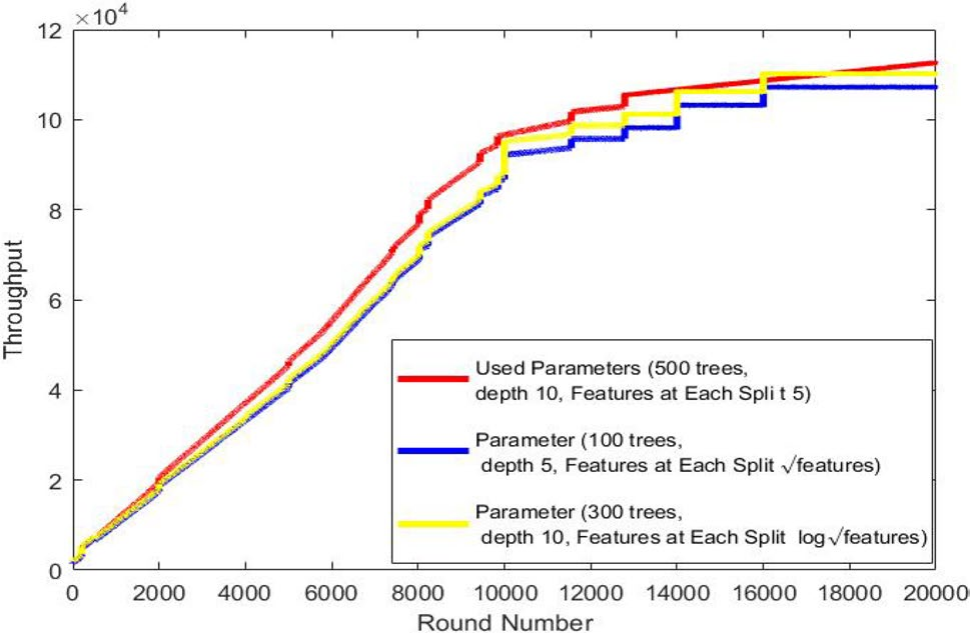
Throughput for each parameter.

Figure 11 compare the effect of different hyperparameters in Throughput.

As can be seen from Fig. 11 the best hyperparameters (500 trees, depth 10, 5 features) achieves better throughput more than the hyperparameters (300 trees, depth 10, Features at Each Split $log \sqrt{features}$) and also more than the hyperparameters (100 trees, depth 5, Features at Each Split features $\sqrt{features}$). This improvement is achieved due to the accurate selection of hyperparameters to help save energy of the sensor node, which leads to keeping it working longer and thus sending data for a longer period which leads to increasing the throughput.

## Conclusion and future work

In this paper, we presented an Activity Recognition adaptive routing protocol for Wireless Body Area Networks (WBANs) that uses Human Activity Recognition (HAR) to improve routing performance. By utilizing a Random Forest algorithm trained on the PAMAP2 dataset, our protocol can recognize user activities and based on it make optimized routing decisions to save energy, avoid network disconnections, and ensure quality service. The choice of random forest as it consume minimum power best fit the nature of WBAN and help to save more energy. Our results showed that the HAR-based routing protocol significantly outperforms the mobTHE protocol in terms of energy efficiency, network stability, and throughput. The protocol effectively extends network lifetime by dynamically adjusting data transmission and resource allocation based on the user’s activity state (e.g., resting or exercising). This adaptive approach reduces energy consumption during low-activity periods and ensures reliable data delivery during high-activity periods.

Overall, our research highlights the potential of combining HAR with adaptive routing to optimize WBAN performance in healthcare and related applications. It demonstrates how activity recognition can be effectively integrated with routing protocols to improve efficiency and reliability in real-world environments.

In the future , we will train a simpler student model using knowledge from the larger teacher model ( Random Forest-based HAR) this is known as Knowledge Distillation (KD) which reduce energy consumption in WBANs while keeping high accuracy. This will make activity recognition and routing faster and more energy-efficient, improving overall network performance.

## Data Availability

The data that support the findings of this study are available from the corresponding author, Enas Selem, upon reasonable request. The data that support the findings of this study (PAMAP2 Physical Activity Monitoring dataset) contains data of 18 different physical activities, performed by 9 subjects wearing 3 inertial measurement units and a heart rate monitor. It is openly available in [UCI Machine Learning Repository”] at https://archive.ics.uci.edu/dataset/231/pamap2+physical+activity+monitoring.
